# Supplementary material for: Dynamics of the MRSA Population in a Chilean Hospital: a Phylogenomic Analysis (2000–2016)
Source: Microbiol Spectr. 2023 Jun 20;11(4):e05351-22. doi: 10.1128/spectrum.05351-22 (PMC10433796; doi:10.1128/spectrum.05351-22)
Supplement: Supplemental file 1 — Supplemental material. Download spectrum.05351-22-s0001.docx, DOCX file, 0.7 MB [file spectrum.05351-22-s0001.docx]

**Supplementary material**


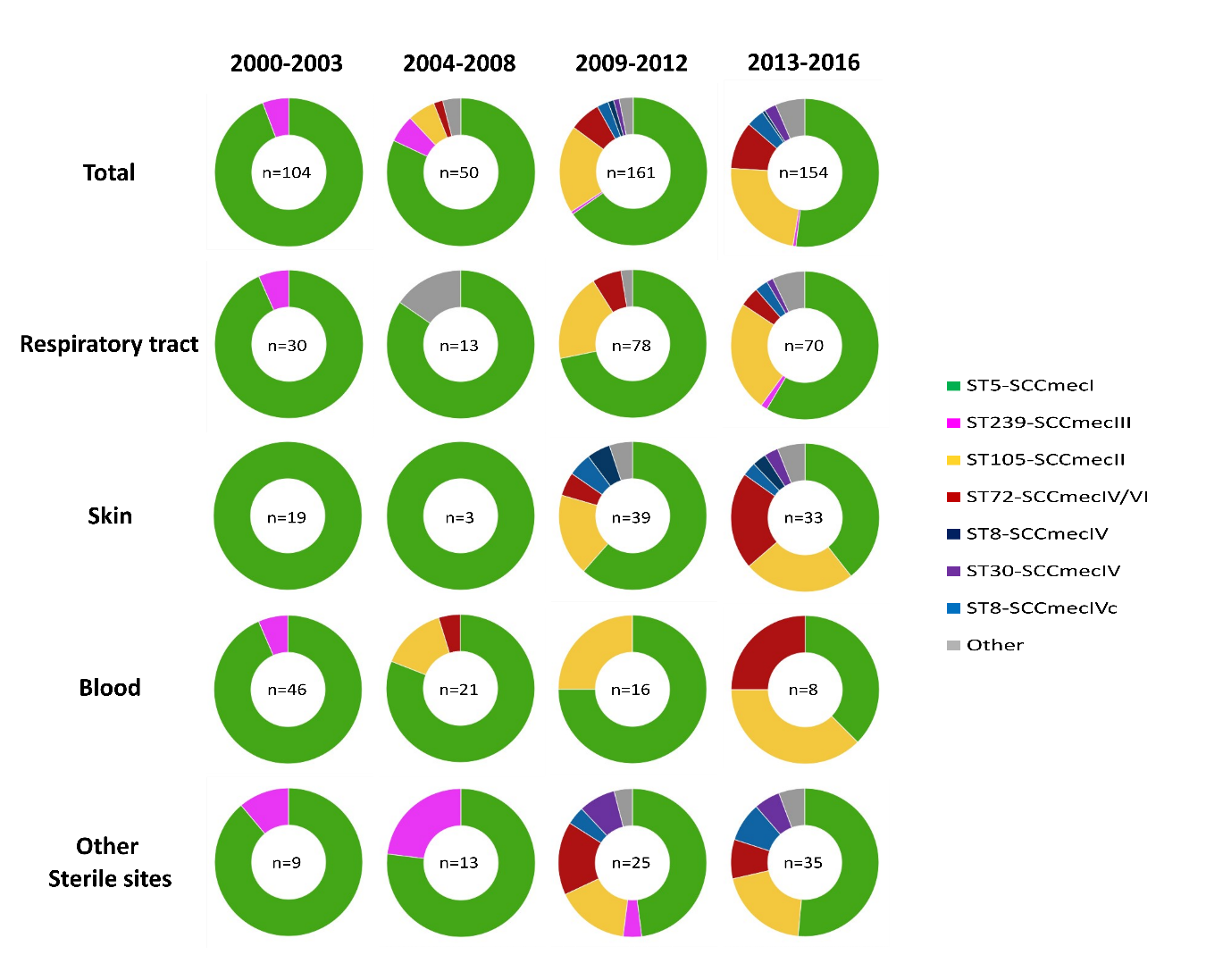


**Supplementary Figure 1. Temporal trends of the most frequent clones by anatomical sites.**
